# 9-(4-Chloro­phen­yl)-4a-hy­droxy-4,4a,5,6,9,9a-hexa­hydro-3*H*-xanthene-1,8(2*H*,7*H*)-dione

**DOI:** 10.1107/S1600536811031977

**Published:** 2011-08-27

**Authors:** Yan Yang, Weicheng Lu, Chaomei Lian, Yulin Zhu

**Affiliations:** a School of Chemistry and Environment, South China Normal University, Guangzhou 510006, People’s Republic of China

## Abstract

In the title compound, C_19_H_19_ClO_4_, the central fused ring and the attached cyclo­hexene ring adopt envelope conformations, while the cyclo­hexane ring adopts a chair conformation. The crystal packing is stabilized by O—H⋯O hydrogen bonds, which link the mol­ecules into a chain along the *b* axis. Weak C—H⋯O bonds also occur.

## Related literature

For the bilogical activity of xanthenes, see: Srividya *et al.* (1996 ▶); Wang *et al.*, (2006 ▶); Kantevari *et al.* (2006 ▶); Reddy *et al.* (2009 ▶); Mehdi *et al.* (2011 ▶); Mo *et al.* (2010 ▶). For the synthesis of related compounds, see: Karade *et al.* (2007 ▶); Luna *et al.* (2009 ▶).
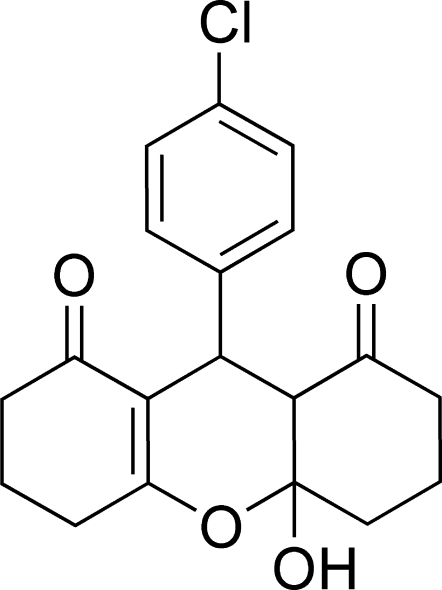


## Experimental

### 

#### Crystal data

C_19_H_19_ClO_4_
*M* *_r_* = 346.79
Monoclinic, 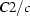

*a* = 25.076 (3) Å
*b* = 12.7715 (13) Å
*c* = 11.3825 (11) Å
β = 110.307 (1)°
*V* = 3418.7 (6) Å^3^
*Z* = 8
Mo *K*α radiation
μ = 0.24 mm^−1^
*T* = 298 K
0.30 × 0.15 × 0.15 mm

#### Data collection

Bruker APEXII area-detector diffractometer
Absorption correction: multi-scan (*SADABS*; Bruker, 2002 ▶) *T*_min_ = 0.931, *T*_max_ = 0.965
8674 measured reflections
3095 independent reflections
1984 reflections with *I* > 2σ(*I*)
*R*_int_ = 0.035

#### Refinement

*R*[*F* ^2^ > 2σ(*F* ^2^)] = 0.042
*wR*(*F* ^2^) = 0.111
*S* = 1.02
3095 reflections
219 parameters
H-atom parameters constrained
Δρ_max_ = 0.17 e Å^−3^
Δρ_min_ = −0.22 e Å^−3^

### 

Data collection: *APEX2* (Bruker, 2004 ▶); cell refinement: *SAINT* (Bruker, 2004 ▶); data reduction: *SAINT*; program(s) used to solve structure: *SHELXS97* (Sheldrick, 2008 ▶); program(s) used to refine structure: *SHELXL97* (Sheldrick, 2008 ▶); molecular graphics: *SHELXTL* (Sheldrick, 2008 ▶); software used to prepare material for publication: *SHELXTL*.

## Supplementary Material

Crystal structure: contains datablock(s) global, I. DOI: 10.1107/S1600536811031977/aa2018sup1.cif

Structure factors: contains datablock(s) I. DOI: 10.1107/S1600536811031977/aa2018Isup2.hkl

Supplementary material file. DOI: 10.1107/S1600536811031977/aa2018Isup3.cml

Additional supplementary materials:  crystallographic information; 3D view; checkCIF report

## Figures and Tables

**Table 1:** Hydrogen-bond geometry (Å, °)

*D*—H⋯*A*	*D*—H	H⋯*A*	*D*⋯*A*	*D*—H⋯*A*
O2—H2⋯O3^i^	0.82	1.84	2.654 (2)	172
C2—H10*A*⋯O1^ii^	0.97	2.52	3.199 (3)	127
C11—H6⋯O4^iii^	0.93	2.56	3.483 (3)	174
C15—H4⋯O1^iv^	0.93	2.58	3.452 (3)	156
C5—H17*A*⋯O3^iv^	0.97	2.51	3.398 (3)	153

Symmetry codes: (i) 
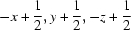
; (ii) 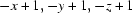; (iii) 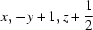; (iv) 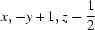.

**Fig. 1.**
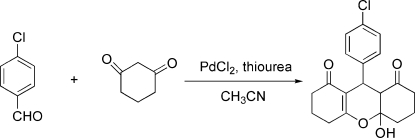
Palladium(II) chloride catalyzed synthesis of the title compound.

**Fig. 2.**
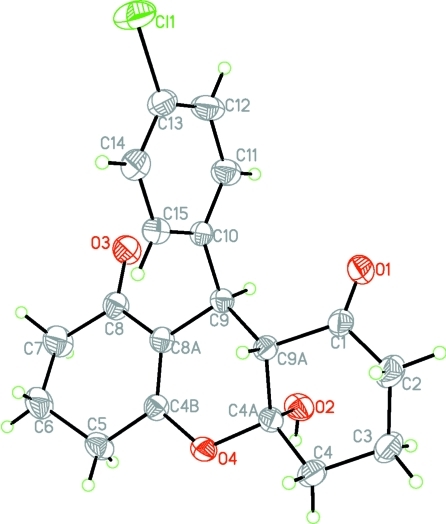
A view of the title compound with the atom numbering scheme. Thermal displacement ellipsoids are drawn at the 50% probability level.

## supplementary crystallographic information

### Comment

Xanthenes are an important class of organic compounds which attract researchers by their spectroscopic and biological properties. Their derivatives had been tested to possess antitumoral, fungicidal, antiinflammatory and bactericidal properties. (Kantevari *et al.*, 2006; Srividya *et al.*, 1996; Wang *et al.*, 2006; Reddy *et al.*, 2009; Mehdi *et al.*, 2011; Mo *et al.*, 2010). A well established method used for the construction of xanthene unit was a tandem Michael reaction between 1,3-cyclohexanedione and benzaldehyde (Luna *et al.*, 2009; Karade *et al.*, 2007). The reaction between 1,3-cyclohexanedione and 4-chlorobenzaldehyde in the presence of thiourea and palladium(II) chloride proceeded to give the title compound with yield 91% (Fig. 1). The main structure of this compound  (Fig. 2) is a derivated xanthenedione fused tricyclo ring with a hydroxyl group at its C4A position. The phenly ring is attached to a tricyclo ring at the C9 position. The central ring and the attached cyclohexene ring adopt an envelope conformation while the cyclohexane ring adopts a chair conformation. The crystal packing is stabilized by O—H···O hydrogen bond which links molecules into a chain along *b* axis. Weak C—H···O bonds were also found in this structure.

### Experimental

A mixture of 1,3-cyclohexanedione (1.12 g, 10 mmol), 4-chloro-benzaldehyde (0.7 g, 5 mmol), thiourea (0.76 g,10 mmol) and palladium (II) chloride (0.0020 mg) was refluxed in anhydrous acetonitrile (12 ml) at 373 K for 10 h. After being cooled to room temperature, the reaction mixture was poured into water. The white precipitate was filtered off with a silica pad, washed twice with anhydrous ethanol, and the filtrate was then dried under vacuum to yield the product in yield of 91% (Fig. 1). Single crystals of the title compound were obtained by slow evaporation from anhydrous ethanol at room temperature to yield colourless, block-shaped crystal.

### Refinement

The H atoms were positioned geometrically and allowed to ride on their parent atoms, with C—H = 0.93–0.98 Å and O—H = 0.82 Å, and *U*_iso_ =1.2 or 1.5U_eq_(parent atom).

### Crystal data

**Table:**

C_19_H_19_ClO_4_	*F*(000) = 1456
*M**_r_* = 346.79	*D*_x_ = 1.348 Mg m^−^^3^
Monoclinic, *C*2/*c*	Mo *K*α radiation, λ = 0.71073 Å
Hall symbol: -C 2yc	Cell parameters from 1769 reflections
*a* = 25.076 (3) Å	θ = 2.4–20.3°
*b* = 12.7715 (13) Å	µ = 0.24 mm^−^^1^
*c* = 11.3825 (11) Å	*T* = 298 K
β = 110.307 (1)°	Block, colourless
*V* = 3418.7 (6) Å^3^	0.30 × 0.15 × 0.15 mm
*Z* = 8	

### Data collection

**Table:**

Bruker APEXII area-detector diffractometer	3095 independent reflections
Radiation source: fine-focus sealed tube	1984 reflections with *I* > 2σ(*I*)
graphite	*R*_int_ = 0.035
φ and ω scans	θ_max_ = 25.3°, θ_min_ = 1.7°
Absorption correction: multi-scan (*SADABS*; Bruker, 2002)	*h* = −30→30
*T*_min_ = 0.931, *T*_max_ = 0.965	*k* = −10→15
8674 measured reflections	*l* = −13→13

### Refinement

**Table:**

Refinement on *F*^2^	Secondary atom site location: difference Fourier map
Least-squares matrix: full	Hydrogen site location: inferred from neighbouring sites
*R*[*F*^2^ > 2σ(*F*^2^)] = 0.042	H-atom parameters constrained
*wR*(*F*^2^) = 0.111	*w* = 1/[σ^2^(*F*_o_^2^) + (0.0479*P*)^2^ + 0.6651*P*] where *P* = (*F*_o_^2^ + 2*F*_c_^2^)/3
*S* = 1.02	(Δ/σ)_max_ < 0.001
3095 reflections	Δρ_max_ = 0.17 e Å^−^^3^
219 parameters	Δρ_min_ = −0.22 e Å^−^^3^
0 restraints	Extinction correction: *SHELXL97* (Sheldrick, 2008), Fc^*^=kFc[1+0.001xFc^2^λ^3^/sin(2θ)]^-1/4^
Primary atom site location: structure-invariant direct methods	Extinction coefficient: 0.0007 (2)

### Special details

**Table:**

Geometry. All s.u.'s (except the s.u. in the dihedral angle between two l.s. planes) are estimated using the full covariance matrix. The cell s.u.'s are taken into account individually in the estimation of s.u.'s in distances, angles and torsion angles; correlations between s.u.'s in cell parameters are only used when they are defined by crystal symmetry. An approximate (isotropic) treatment of cell s.u.'s is used for estimating s.u.'s involving l.s. planes.
Refinement. Refinement of F^2^ against ALL reflections. The weighted R-factor wR and goodness of fit S are based on F^2^, conventional R-factors R are based on F, with F set to zero for negative F^2^. The threshold expression of F^2^ > 2sigma(F^2^) is used only for calculating R-factors(gt) etc. and is not relevant to the choice of reflections for refinement. R-factors based on F^2^ are statistically about twice as large as those based on F, and R- factors based on ALL data will be even larger.

### Fractional atomic coordinates and isotropic or equivalent isotropic displacement parameters (Å^2^)

**Table:**

	*x*	*y*	*z*	*U*_iso_*/*U*_eq_	
Cl1	0.44721 (4)	0.02958 (6)	0.34171 (8)	0.1002 (3)	
O4	0.31880 (6)	0.64755 (11)	0.14155 (12)	0.0466 (4)	
C9	0.34321 (8)	0.45993 (15)	0.29866 (18)	0.0396 (5)	
H7	0.3292	0.4676	0.3686	0.048*	
C1	0.42884 (9)	0.56223 (17)	0.4411 (2)	0.0497 (6)	
C8A	0.29282 (8)	0.47462 (17)	0.17790 (19)	0.0423 (5)	
C10	0.36998 (8)	0.35209 (16)	0.30869 (18)	0.0396 (5)	
C9A	0.38633 (8)	0.54699 (15)	0.30970 (18)	0.0406 (5)	
H8	0.4075	0.5298	0.2543	0.049*	
C4B	0.28472 (8)	0.56314 (18)	0.10889 (19)	0.0428 (5)	
C15	0.39198 (10)	0.31582 (18)	0.2212 (2)	0.0536 (6)	
H4	0.3903	0.3583	0.1537	0.064*	
C4	0.39848 (10)	0.73910 (18)	0.2721 (2)	0.0558 (6)	
H12A	0.3777	0.8040	0.2456	0.067*	
H12B	0.4180	0.7233	0.2141	0.067*	
C13	0.41858 (10)	0.15508 (18)	0.3296 (2)	0.0576 (7)	
C5	0.23679 (9)	0.58061 (19)	−0.0116 (2)	0.0554 (6)	
H17A	0.2504	0.6202	−0.0683	0.066*	
H17B	0.2073	0.6213	0.0041	0.066*	
C8	0.24755 (9)	0.39807 (19)	0.1437 (2)	0.0517 (6)	
C11	0.37342 (10)	0.28731 (19)	0.4084 (2)	0.0541 (6)	
H6	0.3593	0.3104	0.4695	0.065*	
C12	0.39749 (10)	0.1886 (2)	0.4188 (2)	0.0616 (7)	
H1	0.3993	0.1455	0.4860	0.074*	
C2	0.47120 (10)	0.64849 (19)	0.4547 (3)	0.0695 (8)	
H10A	0.4964	0.6297	0.4099	0.083*	
H10B	0.4941	0.6568	0.5425	0.083*	
C3	0.44180 (10)	0.75218 (19)	0.4037 (2)	0.0687 (8)	
H11A	0.4228	0.7783	0.4589	0.082*	
H11B	0.4701	0.8034	0.4020	0.082*	
C14	0.41648 (11)	0.2179 (2)	0.2313 (2)	0.0613 (7)	
H3	0.4314	0.1950	0.1715	0.074*	
C7	0.19886 (10)	0.4081 (2)	0.0210 (2)	0.0743 (8)	
H15A	0.1890	0.3390	−0.0155	0.089*	
H15B	0.1660	0.4358	0.0370	0.089*	
C6	0.21224 (11)	0.4780 (2)	−0.0717 (2)	0.0720 (8)	
H16A	0.1778	0.4910	−0.1426	0.086*	
H16B	0.2393	0.4433	−0.1024	0.086*	
O1	0.42757 (7)	0.50975 (13)	0.52877 (15)	0.0629 (5)	
O2	0.32669 (7)	0.66998 (12)	0.34916 (14)	0.0557 (4)	
H2	0.3049	0.7193	0.3225	0.084*	
O3	0.24715 (7)	0.32685 (13)	0.21722 (14)	0.0634 (5)	
C4A	0.35751 (9)	0.65211 (16)	0.26941 (19)	0.0426 (5)	

### Atomic displacement parameters (Å^2^)

**Table:**

	*U*^11^	*U*^22^	*U*^33^	*U*^12^	*U*^13^	*U*^23^
Cl1	0.1129 (7)	0.0477 (4)	0.1184 (7)	0.0259 (4)	0.0126 (5)	0.0036 (4)
O4	0.0448 (8)	0.0439 (9)	0.0435 (8)	−0.0013 (8)	0.0059 (7)	0.0069 (7)
C9	0.0383 (11)	0.0396 (12)	0.0388 (11)	−0.0018 (10)	0.0107 (9)	0.0008 (9)
C1	0.0461 (13)	0.0374 (13)	0.0534 (14)	0.0104 (11)	0.0018 (11)	−0.0065 (11)
C8A	0.0355 (11)	0.0448 (13)	0.0442 (12)	−0.0033 (10)	0.0106 (10)	0.0000 (11)
C10	0.0373 (11)	0.0370 (12)	0.0387 (11)	−0.0051 (10)	0.0057 (9)	−0.0002 (10)
C9A	0.0378 (11)	0.0357 (12)	0.0445 (12)	0.0006 (10)	0.0095 (10)	−0.0020 (10)
C4B	0.0361 (11)	0.0472 (14)	0.0434 (12)	−0.0042 (11)	0.0116 (10)	−0.0004 (10)
C15	0.0692 (15)	0.0434 (14)	0.0449 (13)	0.0043 (13)	0.0155 (12)	0.0025 (11)
C4	0.0543 (14)	0.0386 (13)	0.0649 (15)	−0.0021 (12)	0.0085 (12)	0.0008 (12)
C13	0.0535 (14)	0.0392 (14)	0.0655 (16)	0.0009 (12)	0.0020 (13)	−0.0013 (13)
C5	0.0433 (13)	0.0641 (16)	0.0502 (13)	0.0027 (12)	0.0053 (11)	0.0110 (12)
C8	0.0430 (13)	0.0560 (15)	0.0514 (13)	−0.0089 (12)	0.0104 (11)	−0.0007 (12)
C11	0.0572 (14)	0.0508 (15)	0.0541 (14)	−0.0007 (12)	0.0191 (12)	0.0088 (12)
C12	0.0616 (15)	0.0510 (15)	0.0655 (16)	−0.0008 (13)	0.0134 (14)	0.0198 (13)
C2	0.0512 (14)	0.0474 (15)	0.0855 (18)	−0.0023 (13)	−0.0070 (13)	−0.0072 (13)
C3	0.0606 (16)	0.0410 (14)	0.0834 (18)	−0.0055 (13)	−0.0015 (14)	−0.0059 (13)
C14	0.0728 (17)	0.0524 (16)	0.0549 (15)	0.0082 (14)	0.0173 (13)	−0.0064 (13)
C7	0.0525 (15)	0.081 (2)	0.0686 (17)	−0.0218 (15)	−0.0054 (13)	0.0077 (15)
C6	0.0617 (16)	0.080 (2)	0.0533 (15)	−0.0147 (15)	−0.0061 (13)	0.0034 (14)
O1	0.0702 (11)	0.0537 (11)	0.0493 (10)	0.0097 (9)	0.0012 (8)	−0.0029 (8)
O2	0.0574 (10)	0.0529 (11)	0.0557 (9)	0.0187 (8)	0.0182 (8)	0.0032 (8)
O3	0.0571 (10)	0.0657 (11)	0.0588 (10)	−0.0254 (9)	0.0089 (8)	0.0072 (9)
C4A	0.0409 (11)	0.0400 (13)	0.0417 (12)	0.0045 (10)	0.0079 (10)	0.0006 (10)

### Geometric parameters (Å, °)

**Table:**

Cl1—C13	1.742 (2)	C13—C14	1.362 (3)
O4—C4B	1.345 (2)	C13—C12	1.367 (3)
O4—C4A	1.443 (2)	C5—C6	1.507 (3)
C9—C10	1.519 (3)	C5—H17A	0.9700
C9—C8A	1.522 (3)	C5—H17B	0.9700
C9—C9A	1.525 (3)	C8—O3	1.238 (3)
C9—H7	0.9800	C8—C7	1.508 (3)
C1—O1	1.212 (3)	C11—C12	1.385 (3)
C1—C2	1.501 (3)	C11—H6	0.9300
C1—C9A	1.518 (3)	C12—H1	0.9300
C8A—C4B	1.351 (3)	C2—C3	1.529 (3)
C8A—C8	1.445 (3)	C2—H10A	0.9700
C10—C15	1.375 (3)	C2—H10B	0.9700
C10—C11	1.383 (3)	C3—H11A	0.9700
C9A—C4A	1.518 (3)	C3—H11B	0.9700
C9A—H8	0.9800	C14—H3	0.9300
C4B—C5	1.494 (3)	C7—C6	1.507 (3)
C15—C14	1.381 (3)	C7—H15A	0.9700
C15—H4	0.9300	C7—H15B	0.9700
C4—C4A	1.506 (3)	C6—H16A	0.9700
C4—C3	1.524 (3)	C6—H16B	0.9700
C4—H12A	0.9700	O2—C4A	1.400 (2)
C4—H12B	0.9700	O2—H2	0.8200
			
C4B—O4—C4A	116.83 (16)	O3—C8—C8A	120.3 (2)
C10—C9—C8A	112.73 (16)	O3—C8—C7	119.6 (2)
C10—C9—C9A	111.88 (16)	C8A—C8—C7	120.0 (2)
C8A—C9—C9A	109.10 (16)	C10—C11—C12	121.1 (2)
C10—C9—H7	107.6	C10—C11—H6	119.5
C8A—C9—H7	107.6	C12—C11—H6	119.5
C9A—C9—H7	107.6	C13—C12—C11	119.4 (2)
O1—C1—C2	122.8 (2)	C13—C12—H1	120.3
O1—C1—C9A	122.0 (2)	C11—C12—H1	120.3
C2—C1—C9A	115.2 (2)	C1—C2—C3	111.47 (19)
C4B—C8A—C8	117.64 (19)	C1—C2—H10A	109.3
C4B—C8A—C9	122.58 (19)	C3—C2—H10A	109.3
C8—C8A—C9	119.22 (18)	C1—C2—H10B	109.3
C15—C10—C11	117.8 (2)	C3—C2—H10B	109.3
C15—C10—C9	121.93 (19)	H10A—C2—H10B	108.0
C11—C10—C9	120.3 (2)	C4—C3—C2	111.3 (2)
C1—C9A—C4A	106.39 (16)	C4—C3—H11A	109.4
C1—C9A—C9	114.20 (18)	C2—C3—H11A	109.4
C4A—C9A—C9	111.72 (16)	C4—C3—H11B	109.4
C1—C9A—H8	108.1	C2—C3—H11B	109.4
C4A—C9A—H8	108.1	H11A—C3—H11B	108.0
C9—C9A—H8	108.1	C13—C14—C15	119.4 (2)
O4—C4B—C8A	123.84 (18)	C13—C14—H3	120.3
O4—C4B—C5	111.45 (19)	C15—C14—H3	120.3
C8A—C4B—C5	124.7 (2)	C6—C7—C8	113.2 (2)
C10—C15—C14	121.6 (2)	C6—C7—H15A	108.9
C10—C15—H4	119.2	C8—C7—H15A	108.9
C14—C15—H4	119.2	C6—C7—H15B	108.9
C4A—C4—C3	110.44 (19)	C8—C7—H15B	108.9
C4A—C4—H12A	109.6	H15A—C7—H15B	107.8
C3—C4—H12A	109.6	C7—C6—C5	110.6 (2)
C4A—C4—H12B	109.6	C7—C6—H16A	109.5
C3—C4—H12B	109.6	C5—C6—H16A	109.5
H12A—C4—H12B	108.1	C7—C6—H16B	109.5
C14—C13—C12	120.8 (2)	C5—C6—H16B	109.5
C14—C13—Cl1	120.3 (2)	H16A—C6—H16B	108.1
C12—C13—Cl1	119.0 (2)	C4A—O2—H2	109.5
C4B—C5—C6	111.0 (2)	O2—C4A—O4	109.33 (16)
C4B—C5—H17A	109.4	O2—C4A—C4	113.13 (18)
C6—C5—H17A	109.4	O4—C4A—C4	105.39 (16)
C4B—C5—H17B	109.4	O2—C4A—C9A	105.09 (17)
C6—C5—H17B	109.4	O4—C4A—C9A	110.61 (16)
H17A—C5—H17B	108.0	C4—C4A—C9A	113.34 (17)
			
C10—C9—C8A—C4B	−136.9 (2)	C9—C8A—C8—C7	−176.7 (2)
C9A—C9—C8A—C4B	−12.0 (3)	C15—C10—C11—C12	−0.9 (3)
C10—C9—C8A—C8	51.9 (3)	C9—C10—C11—C12	179.75 (19)
C9A—C9—C8A—C8	176.86 (18)	C14—C13—C12—C11	0.4 (4)
C8A—C9—C10—C15	59.2 (3)	Cl1—C13—C12—C11	−178.49 (17)
C9A—C9—C10—C15	−64.2 (2)	C10—C11—C12—C13	0.5 (4)
C8A—C9—C10—C11	−121.5 (2)	O1—C1—C2—C3	−124.8 (3)
C9A—C9—C10—C11	115.1 (2)	C9A—C1—C2—C3	53.5 (3)
O1—C1—C9A—C4A	122.9 (2)	C4A—C4—C3—C2	53.2 (3)
C2—C1—C9A—C4A	−55.4 (2)	C1—C2—C3—C4	−50.4 (3)
O1—C1—C9A—C9	−0.8 (3)	C12—C13—C14—C15	−0.9 (4)
C2—C1—C9A—C9	−179.16 (18)	Cl1—C13—C14—C15	177.94 (18)
C10—C9—C9A—C1	−72.7 (2)	C10—C15—C14—C13	0.5 (4)
C8A—C9—C9A—C1	161.85 (17)	O3—C8—C7—C6	−164.5 (2)
C10—C9—C9A—C4A	166.48 (17)	C8A—C8—C7—C6	18.8 (4)
C8A—C9—C9A—C4A	41.1 (2)	C8—C7—C6—C5	−49.6 (3)
C4A—O4—C4B—C8A	−14.8 (3)	C4B—C5—C6—C7	50.6 (3)
C4A—O4—C4B—C5	164.42 (18)	C4B—O4—C4A—O2	−70.6 (2)
C8—C8A—C4B—O4	168.96 (19)	C4B—O4—C4A—C4	167.55 (17)
C9—C8A—C4B—O4	−2.3 (3)	C4B—O4—C4A—C9A	44.7 (2)
C8—C8A—C4B—C5	−10.2 (3)	C3—C4—C4A—O2	60.3 (2)
C9—C8A—C4B—C5	178.6 (2)	C3—C4—C4A—O4	179.72 (19)
C11—C10—C15—C14	0.4 (3)	C3—C4—C4A—C9A	−59.2 (3)
C9—C10—C15—C14	179.70 (19)	C1—C9A—C4A—O2	−66.0 (2)
O4—C4B—C5—C6	158.98 (19)	C9—C9A—C4A—O2	59.2 (2)
C8A—C4B—C5—C6	−21.8 (3)	C1—C9A—C4A—O4	176.08 (17)
C4B—C8A—C8—O3	−164.9 (2)	C9—C9A—C4A—O4	−58.7 (2)
C9—C8A—C8—O3	6.7 (3)	C1—C9A—C4A—C4	58.0 (2)
C4B—C8A—C8—C7	11.8 (3)	C9—C9A—C4A—C4	−176.76 (17)

### Hydrogen-bond geometry (Å, °)

**Table:**

*D*—H···*A*	*D*—H	H···*A*	*D*···*A*	*D*—H···*A*
O2—H2···O3^i^	0.82	1.84	2.654 (2)	172.
C2—H10A···O1^ii^	0.97	2.52	3.199 (3)	127.
C11—H6···O4^iii^	0.93	2.56	3.483 (3)	174.
C15—H4···O1^iv^	0.93	2.58	3.452 (3)	156.
C5—H17A···O3^iv^	0.97	2.51	3.398 (3)	153.

Symmetry codes: (i) −*x*+1/2, *y*+1/2, −*z*+1/2; (ii) −*x*+1, −*y*+1, −*z*+1; (iii) *x*, −*y*+1, *z*+1/2; (iv) *x*, −*y*+1, *z*−1/2.
